# Fine-mapping markers of lung cancer susceptibility in a sub-region of chromosome 19q13.3 among Chinese

**DOI:** 10.18632/oncotarget.9279

**Published:** 2016-05-10

**Authors:** Jiaoyang Yin, Huiwen Wang, Ulla Vogel, Chunhong Wang, Yegang Ma, Wei Hou, Ying Zhang, Li Guo, Xinxin Li

**Affiliations:** ^1^ Key Laboratory of Environment and Population Health of Liaoning Education Ministry (Shenyang Medical College), Shenyang, Liaoning Province, People's Republic of China; ^2^ National Research Centre for The Working Environment, Lerso Parkalle, Copenhagen O, Denmark; ^3^ Department of Thoracic Surgery, Liaoning Cancer Hospital, Shenyang, Liaoning Province, People's Republic of China; ^4^ Department of Pathology, School of Basic Medical Sciences, Health Science Center, Peking University, Beijing, People's Republic of China

**Keywords:** Chr19q13.3, ERCC2 and PPP1R13L and CD3EAP and ERCC1, fine-mapping, lung cancer, Chinese

## Abstract

Linkage disequilibrium-mapping studies in Caucasians have indicated anassociation of Chr19q13.3 sub-region spanning *ERCC2*, *PPP1R13L*, *CD3EAP* and *ERCC1* with several cancers. To refine the region of association and identify potential causal variations among Asians, we performed a fine-mapping study using 32 (39) SNPs in a 71.654kb sub-region. The study included 384 Chinese lung cancer cases and 387 controls. Seven closely situated SNPs showed significant associations with lung cancer risk in five different genetic models of single-locus associations (adjusted for smoking duration). These were *PPP1R13L* rs1970764 [OR (95% CI) = 1.58 (1.09-2.29), *P* = 0.014] in a recessive model and *PPP1R13L* rs1005165 [OR (95% CI) = 1.25 (1.01-1.54), *P* = 0.036], *CD3EAP* rs967591 [OR (95% CI) = 1.40 (1.13-1.75), *P* = 0.0023], rs735482 [OR (95% CI) = 1.29 (1.03-1.61), *P* = 0.026], rs1007616 [OR (95% CI) = 0.78 (0.61-1.00), *P* = 0.046], and rs62109563 [OR (95% CI) = 1.28 (1.03-1.59), *P* = 0.024] in a log-additive model and *ERCC1* rs3212965 [OR (95% CI) = 0.70 (0.52-0.94), *P* = 0.019] in an over-dominant model. Six-haplotype blocks were determined in the sub-region. Using an alternative approach where we performed a haplotype analysis of all significant polymorphisms, rs1970764 was found to be most consistently associated with lung cancer risk. The combined data suggest that the sub-region with the strongest association to lung cancer susceptibility might locate to the 23.173kb from *PPP1R13L* intron8 rs1970764 to rs62109563 3′ to *CD3EAP*. Limited risk loci and span on lung cancer in this sub-region are initially defined among Asians.

## INTRODUCTION

**Figure 1:**
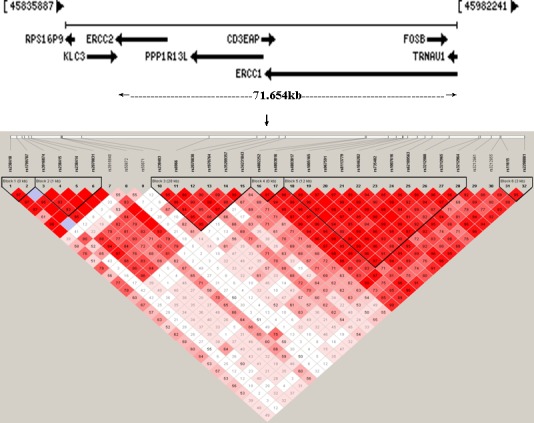
Schematic presentations of the studied genomic region 19q13.3 Upper panel: Gene locations with HUGO gene nomenclature on the chromosome sub-region 19q13.3 are shown. The arrow length represents the extent of the gene and the arrowhead indicates the transcription direction (From: http://www.ncbi.nlm.nih.gov/)(Adopted time: 2013-10-31). The distributed extent of 39 SNPs related to *ERCC2*, *PPP1R13L, CD3EAP* and *ERCC1* in current study was 71.654kb. Lower panel: D′ LD map and LD plot of 32 SNPs covering *ERCC2* (SNP1~10), *PPP1R13L* (SNP11~19)*, CD3EAP* (SNP20~25) and *ERCC1* (SNP26~32) on chromosome 19q13.3 from this Chinese lung cancer study. SNPs in 3′ to 5′ order as indicated in Table 4. Six blocks were detected by Haploview 4.2. The criteria of block partition were based on 95% confidence interval bounds of D′ values. The digit in the boxes D′ value (e.g. 99 means 0.99; 1 means 0.01; empty boxes means 1.0). Deep red boxes designate strong evidence of LD. Light red boxes designate uninformative. White boxes designate strong evidence of recombination.

The candidate sub-region of chromosome 19q13.3 includes four genes. From 3′→5′, they are *ERCC2/XPD* (excision repair cross-complementing rodent repair deficiency, complementation group 2/xeroderma pigmentosum complementary group D), *PPP1R13L/IASPP/RAI* [protein phosphatase 1, regulatory (inhibitor) subunit 13 like/Inhibitory member of the ASPP family/RelA-associated inhibitor], *CD3EAP/ASE-1* [CD3e molecule, epsilon-associated protein/antisense to ERCC1)], and *ERCC1* (excision repair cross-complementing rodent repair deficiency, complementation group 1) (Figure 1). *ERCC2* and *ERCC1* are involved in DNA repair while *PPP1R13L* and *CD3EAP* participate in apoptosis and rRNA transcription, respectively [1, 2]. Genetically determined changes in activity of any of the four genes may play vital roles in carcinogenesis.

Lung cancer is a leading cause of death worldwide [3]. Genetically determined susceptibility may contribute to carcinogenesis, possibly through gene-environment interactions. Three linkage disequilibrium (LD)-mapping studies in Caucasian populations have identified the sub-region encompassing *ERCC2*, *PPP1R13L*, *CD3EAP* and *ERCC1* within chromosome 19q13.3 as being associated with risk of basal cell carcinoma, breast cancer and with multiple myeloma prognosis [4, 5, 1, 6]. Fine-mapping of cancer susceptibility related to the four genes at this chromosome region is still pending in Asian populations. Since the allele frequencies of a number of polymorphisms are very different among Caucasians and Asians, fine-mapping of the region in Asians may provide a tool for identification of the causal genetic variants.

In previous studies, we have examined all the commonly occurring single nucleotide polymorphisms (SNP) of the four genes at chromosome 19q13.3 in relation to lung cancer risk [7–10]. In order to further refine genetic variant location of lung cancer susceptibility at the same region in Chinese populations, we here used a dense fine-mapping strategy and performed extensive genotyping of SNPs.

## RESULTS

The current study group included 384 lung cancer cases and 387 cancer-free controls. Lung cancer cases had significantly higher occurrence of cancer family history [OR (95% CI) = 11.70 (4.62-29.66), *P* < 0.0001] and longer smoking history (> 20 years) (*P* < 0.0001) compared to the control group. There was no notably statistically significant difference between cases and controls for mean age and gender. There were more males than females among cases (Table 1).

**Table 1:** Basic characteristics of lung cancer cases and controls

	Lung cancer cases *n* (%)	Controls n (%)	OR (95% CI)	*P*-value
	**(*n* = 384)**	**(*n* = 387)**		
**Age (years)**				
**Mean (±SD)**	58 (±11)	58 (±11)		0.91^a^
≤40	21 (5.5)	20 (5.2)		
41–50	70 (18.2)	83 (21.4)		
51–60	125 (32.6)	116 (30.0)		0.69^b^
>60	168 (43.8)	168 (43.4)		
**Gender**				
Male	273 (71.1)	276 (71.3)		
Female	111 (28.9)	111 (28.7)		0.95^b^
**Family history** ^c^				
No^d^	333 (86.7)	382 (98.7)	1.0	
Yes	51 (13.3)	5 (1.3)	11.70 (4.62-29.66)	**<0.0001** ^b^
**Smoking history**				
Never^d^	133 (34.6)	206 (53.2)	1.0	
≤20 (years)	67 (17.4)	73 (18.9)	1.42 (0.96-2.11)	**<0.0001** ^b^
>20 (years)	184 (47.9)	108 (27.9)	2.64 (1.91-3.64)	

a For t' test

b For χ^2^ test (two-sided), boldface means statistical significance

c Family history of cancer

d No family history group and Never smoking group as reference category, respectively

Thirty-nine polymorphisms were genotyped in the chromosome 19q13.3 region encompassing the 4 genes. Genotyping revealed no variant alleles for rs10418623, rs3212967, and rs3212950. All other polymorphisms were in Hardy-Weinberg equilibrium among controls except rs2097215, rs8112723, rs201704 and rs3212986. So, these 7 SNPs were excluded leaving 32 SNPs for the subsequent evaluation.

Thirty-two SNPs in 19q13.3 were analyzed using five different genetic models in relation to lung cancer risk adjusting for smoking duration (Table 2). *PPP1R13L* rs1970764, rs1005165, *CD3EAP* rs967591, rs735482, rs1007616, rs62109563, and *ERCC1* rs3212965 were all associated with lung cancer risk in at least one of the models. The associations were evaluated on the basis of AIC (Akaike's Information Criterion) for different genetic models (to choose the smallest values). Six SNPs, *PPP1R13L* rs1970764 [OR, Odd Ratio (95% CI,) = 1.58 (1.09-2.29), *P* = 0.014] in the recessive model and *PPP1R13L* rs1005165 [OR (95% CI) = 1.25 (1.01-1.54), *P* = 0.036], *CD3EAP* rs967591 [OR (95% CI) = <1.40 (1.13-1.75), *P* = 0.0023], rs735482 [OR (95% CI) = 1.29 (1.03-1.61), *P* = 0.026], rs1007616 [OR (95% CI) = 0.78 (0.61-1.00), *P* = 0.046], and rs62109563 [OR (95% CI) = 1.28 (1.03-1.59), *P* = 0.024] in the log-additive model, were associated with increased lung cancer risk. *ERCC1* rs3212965 [OR (95% CI) = 0.70 (0.52-0.94), *P* = 0.019] was associated with lowered lung cancer risk in the over-dominant model. The association of *CD3EAP* rs967591 [OR (95% CI) = 1.40 (1.13-1.75), *P* = 0.0023] with lung cancer risk was the strongest using the log-additive model.

**Table 2:** Single analysis for lung cancer association of 32 SNPs on Chromosome 19q13.3 in five genetic models^a^, OR (95%CI) adjusted for smoking duration

Gene/rs	Ca/Co	Co-dominant	Dominant	Recessive	Over-dominant	Log-additive
		(AB vs AA)/(BB vs AA)/*P*	(AB+BB vs AA)/*P*	(BB vs AA+AB)/*P*	(AB vs AA+BB)/*P*	(− −)/*P*
***ERCC2***						
rs238418	378/384	1.05 (0.77-1.43)/1.08 (0.66-1.76)/0.93	1.06 (0.79-1.42)/0.7	11.05 (0.66-1.67)/0.84	1.04 (0.77-1.39)/0.8	1.04 (0.84-1.30)/0.71
rs1799787	320/344	1.10 (0.68-1.81)/0.92 (0.12-6.88)/0.92	1.09 (0.68-1.77)/0.7	10.91 (0.12-6.79)/0.93	1.11 (0.68-1.81)/0.69	1.08 (0.69-1.68)/0.75
rs3916874	331/346	0.97 (0.69-1.38)/0.42 (0.16-1.15)/0.21	0.90 (0.64-1.26)/0.5	40.43 (0.16-1.15)/0.08	1.01 (0.71-1.42)/0.98	0.85 (0.63-1.14)/0.28
rs238415	381/382	1.00 (0.71-1.40)/1.14 (0.75-1.73)/0.78	1.04 (0.76-1.43)/0.81	1.14 (0.79-1.63)/0.48	0.95 (0.71-1.27)/0.72	1.06 (0.86-1.31)/0.58
rs238414	377/383	0.93 (0.66-1.31)/0.80 (0.52-1.23)/0.58	0.89 (0.64-1.24)/0.49	0.84 (0.58-1.21)/0.34	1.02 (0.76-1.37)/0.88	0.90 (0.73-1.11)/ 0.32
rs2070831	381/386	1.13 (0.84-1.54)/0.76 (0.41-1.41)/0.4	1.08 (0.80-1.44)/0.62	0.72 (0.39-1.32)/0.28	1.17 (0.87-1.57)/0.3	1.00 (0.79-1.26)/0.98
rs3916840	329/348	1.05 (0.61-1.80)/NA (0.00-NA)/0.61	1.07 (0.63-1.84)/0.8	NA (0.00-NA)/0.33	1.04 (0.61-1.80)/0.88	1.10 (0.65-1.86)/0.72
rs50872	381/385	1.18 (0.87-1.60)/1.35(0.57-3.20)/0.49	1.19 (0.88-1.61)/0.25	1.27 (0.54-3.00)/0.58	1.16 (0.86-1.58)/0.33	1.17 (0.90-1.53)/0.24
rs50871	310/346	0.91 (0.66-1.27)/1.23 (0.64-2.36)/0.66	0.95 (0.70-1.31)/0.78	1.27 (0.67-2.41)/0.46	0.89 (0.65-1.24)/0.5	1.01 (0.78-1.30)/0.94
rs238403	319/331	0.88 (0.61-1.29)/ 0.94(0.59-1.48)/0.81	0.90 (0.63-1.28)/0.55	1.01 (0.69-1.50)/0.94	0.91 (0.66-1.25)/0.56	0.96 (0.76-1.21)/0.73
***PPP1R13L***						
rs6966	291/300	1.03 (0.69-1.55)/1.17 (0.72-1.89)/0.8	1.07 (0.73-1.57)/0.72	1.14 (0.77-1.70)/0.52	0.96 (0.69-1.34)/0.82	1.08 (0.85-1.37)/0.54
rs2070830	381/384	1.13 (0.83-1.52)/1.25 (0.69-2.25)/0.63	1.14 (0.85-1.53)/0.37	1.18 (0.67-2.09)/0.57	1.10 (0.82-1.47)/0.54	1.12 (0.89-1.42)/0.34
rs1970764	352/360	1.09 (0.76-1.56)/**1.67 (1.07-2.61)/0.046**^b^	1.23 (0.87-1.74)/0.24	**1.58 (1.09-2.29)/0.014**^b^	0.86(0.64-1.16)/0.33	**1.28 (1.03-1.60)/0.026**^b^
rs35209357	381/384	1.19 (0.88-1.61)/1.54 (0.90-2.66)/0.23	1.24 (0.92-1.66)/0.15	1.42 (0.84-2.38)/0.19	1.11 (0.83-1.49)/0.49	1.22 (0.97-1.53)/0.089
rs34231843	379/383	1.14 (0.84-1.54)/1.68 (0.97-2.90)/0.17	1.21 (0.90-1.62)/0.21	1.58 (0.93-2.67)/0.089	1.05 (0.78-1.40)/0.76	1.23 (0.97-1.54)/0.083
rs4802252	330/335	0.80 (0.57-1.12)/0.88 (0.44-1.77)/0.42	0.81 (0.59-1.12)/0.2	0.96 (0.49-1.90)/0.91	0.81 (0.58-1.12)/0.2	0.86 (0.66-1.12)/0.27
rs4803816	380/383	0.75 (0.55-1.03)/0.80 (0.42-1.52)/0.18	0.76 (0.56-1.02)/0.067	0.89 (0.47-1.67)/0.72	0.77 (0.56-1.04)/0.087	0.82 (0.64-1.04)/0.1
rs4803817	330/335	0.73 (0.53-1.02)/0.74(0.42-1.29)/0.15	0.73 (0.53-1.01)/0.053	0.86 (0.51-1.47)/0.59	0.77 (0.56-1.06)/0.11	0.81 (0.63-1.03)/0.084
rs1005165	379/381	1.20 (0.86-1.68)/**1.57(1.03-2.40)**^b^/0.11	1.30 (0.94-1.78)/0.11	1.40 (0.97-2.02)/0.068	1.00 (0.75-1.34)/1	**1.25 (1.01-1.54)/0.036**^b^
***CD3EAP***						
rs967591	352/360	**1.48 (1.05-2.09)/1.93(1.23-3.02)/0.0088**^b^	**1.59 (1.15-2.20)/0.0049**^b^	**1.52 (1.02-2.27)/0.036**^b^	1.17 (0.87-1.59)/0.3	**1.40 (1.13-1.75)/0.0023**^b^
rs8113779	379/384	1.19 (0.85-1.66)/1.52 (1.00-2.33)/0.15	1.27 (0.93-1.75)/0.14	1.37 (0.95-1.98)/0.091	1.00 (0.75-1.34)/0.99	1.23 (1.00-1.52)/0.052
rs1046282	330/335	0.74 (0.53-1.03)/0.78 (0.45-1.35)/0.18	0.74 (0.54-1.02)/0.066	0.90 (0.53-1.53)/0.71	0.77 (0.56-1.06)/0.11	0.82 (0.65-1.05)/0.12
rs735482	330/335	1.28 (0.89-1.85)/**1.66 (1.06-2.62)**^b^/0.084	1.38 (0.98-1.96)/0.067	1.42 (0.97-2.09)/0.074	1.03 (0.76-1.41)/0.84	**1.29 (1.03-1.61)/0.026**^b^
rs1007616	377/384	0.75 (0.55-1.03)/0.66 (0.34-1.25)/0.13	**0.74 (0.55-1.00)/0.048**^b^	0.73 (0.39-1.38)/0.33	0.78 (0.58-1.06)/0.12	**0.78 (0.61-1.00)/0.046**^b^
rs62109563	381/385	1.30 (0.95-1.78)/**1.63 (1.02-2.58)**^b^/0.078	**1.36 (1.01-1.84)/0.042**^b^	1.40 (0.92-2.15)/0.12	1.14 (0.85-1.53)/0.37	**1.28 (1.03-1.59)/0.024**^b^
***ERCC1***						
rs3212980	330/335	0.76 (0.55-1.06)/0.79 (0.45-1.37)/0.25	0.77 (0.56-1.05)/0.095	0.90 (0.53-1.53)/0.71	0.79 (0.58-1.09)/0.15	0.84 (0.66-1.07)/0.15
rs3212965	379/383	**0.70 (0.51-0.95)**^b^/0.98 (0.59-1.63)/0.063	**0.75 (0.56-1.00)/0.049**^b^	0.87 (0.70-1.09)/0.22	**0.70 (0.52-0.94)/0.019**^b^	0.87 (0.70-1.09)/0.22
rs3212964	330/335	1.05 (0.73-1.51)/1.42 (0.90-2.23)/0.28	1.14 (0.81-1.61)/0.44	1.37 (0.92-2.03)/0.12	0.92 (0.67-1.25)/0.59	1.17 (0.94-1.47)/0.16
rs3212961	330/335	1.07 (0.73-1.57)/1.42 (0.91-2.22)/0.25	1.17 (0.82-1.68)/0.39	1.36 (0.94-1.96)/0.1	0.90 (0.66-1.23)/0.51	1.19 (0.95-1.49)/0.13
rs3212955	381/387	1.01 (0.75-1.36)/1.15 (0.64-2.05)/0.89	1.03 (0.77-1.37)/0.86	1.15 (0.65-2.01)/0.63	0.99 (0.74-1.32)/0.94	1.04 (0.82-1.31)/0.74
rs11615	357/378	0.86 (0.63-1.19)/0.82 (0.43-1.57)/0.6	0.86 (0.63-1.16)/0.32	0.86 (0.46-1.63)/0.65	0.88 (0.64-1.20)/0.42	0.88 (0.69-1.13)/0.32
rs2298881	330/335	1.19 (0.84-1.69)/1.40 (0.88-2.23)/0.34	1.24 (0.89-1.73)/0.2	1.26 (0.83-1.91)/0.28	1.06 (0.78-1.45)/0.7	1.18 (0.94-1.49)/0.14

a Dominant model: AB(Heterozygote) + BB(Homozygous variant-type) versus AA(Homozygous wild-type) Recessive model: BB versus AA+AB Co-dominant model: AB versus AA or BB versus AA Over-dominant model: AB versus AA+BB Log-additive model: Analysis of trend where AA is ‘0’, AB is ‘1’ and BB is ‘2’

b Boldface means statistical significance

**Figure 2:**
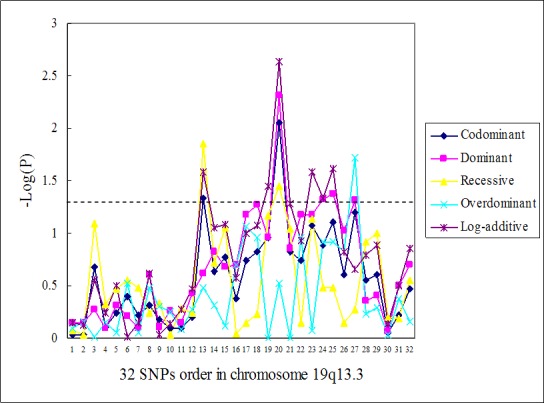
Association of single SNPs with lung cancer for five genetic models, adjusted by smoking duration. Data from Table 2 32 SNPs in 3′ to 5′ order on chromosome 19q13.3 as indicated in Table 4. A dotted line indicates *P*-value of -Log (0.05).

The log transformed *P*-values from the associations between the 32 single SNPs and lung cancer risk in the five genetic models are illustrated in Figure 2. The 7 closely situated markers from *PPP1R13L* rs1970764 (thirteenth SNP) to *ERCC1* rs3212965 (twenty-seventh SNP) constitute a risk sub-region of 29.707kb on chromosome 19q13.3. This sub-region encompasses three genes *PPP1R13L, CD3EAP* and *ERCC1*. The SNP with the most statistically significant association with lung cancer risk was *CD3EAP* rs967591 (twentieth SNP). This SNP was statistically significantly associated with lung cancer risk in four different models (adjusted for smoking duration) (Table 2).

The analysis of single-SNP associations with lung cancer risk was further stratified by smoking duration. We have previously reported that carriers of genotypes of rs967591 (G > A) AA, rs735482 (A > C) AC and CC, rs3212961 (C > A) CA and AA, rs2298881 (C > A) CA and AA were at increased risk among the heavy smokers (> 20 years). There was interaction between rs3212961 and smoking duration (*P*_trend_ = 0.03) [11, 10, 8]. In this study, we re-confirmed the aforementioned results and furthermore showed that carriers of rs1005165 (C > T) TT and CT [TT versus CC, OR (95% CI) = 2.24 (1.08-4.65), CT versus CC, OR (95% CI) = 1.73 (1.02-2.93)] or rs8113779 (G > T) TT [TT versus GG, OR(95% CI) = 2.20 (1.06-4.56)] in this sub-region were at increased lung cancer risk among heavy smokers (> 20 years of smoking) (data not shown).

Linkage disequilibrium and haplotype block structure of the 32 SNPs in the genes *ERCC2, PPP1R13L, CD3EAP* and *ERCC1* are illustrated in Figure 1. Six haplotype blocks were identified based on 95% confidence interval bounds of D′ values (Figure 1). Global test of the haplotype distribution between cases and controls showed statistically different haplotype distribution in block 5 (global *P* = 0.011). Moreover, a protective haplotype ACGGTATTACG spanning 11 SNPs (encompassing the minor allele of rs1007616 and the major alleles of remaining 10 SNPs) of *PPP1R13L, CD3EAP* and *ERCC1* in this block [OR (95%CI) = 0.72 (0.53- 0.97), *P* = 0.032, adjusted for smoking duration] was detected after haplotypes with frequency < 0.03 in both cases and controls were excluded (Supplementary Table 1).

In a second approach used to identify causative polymorphisms, linkage was determined between the seven SNPs which were associated with lung cancer risk (rs1970764, rs1005165, rs967591, rs735482, rs1007616, rs62109563, rs3212965) (Supplementary Figure 1). Rs967591, rs735482 and rs1005165 were in tight linkage with high r^2^ values (Supplementary Figure 1). Therefore, rs735482 and rs1005165 were excluded from further analysis. Haplotype analysis of rs1970764, rs967591, rs1007616, rs62109563, and rs3212965 is shown in Table 3. The *P*-value of the global haplotype association was 0.023. The second most frequent haplotype encompassing the variant alleles of rs1970764, rs967591, and rs62109563 was associated with 1.44-fold (95% CI = 1.05-1.97, *P* = 0.022) increased risk of lung cancer. The sixth haplotype associated with a non-statistically significant 1.8-fold increased risk of lung cancer (95% CI = 0.92-3.54, *P* = 0.088) also included the variant allele of rs1970764 and in addition, the variant allele of rs3212965. Thus, this analysis points to rs1970764 as being most consistently associated with lung cancer risk.

**Table 3:** Haplotype association in 5 significant SNPs, adjusted by smoking duration^a^^,^^b^

Hap	rs1970764	rs967591	rs100761	rs62109563	rs3212965	Control frequency	Case frequency	OR (95% CI)	*P*-value
1	A	G	C	T	T	0.2731	0.2473	1.00	---
2	G	A	C	C	C	0.217	0.2615	**1.44 (1.05 - 1.97)**^c^	**0.022**^c^
3	G	G	T	T	C	0.1198	0.1262	1.12 (0.76 - 1.66)	0.57
4	A	G	T	T	C	0.1123	0.0876	0.85 (0.54 - 1.33)	0.47
5	A	A	C	C	C	0.0879	0.0995	1.33 (0.82 - 2.15)	0.25
6	G	G	C	T	T	0.0336	0.0509	1.80 (0.92 - 3.54)	0.088
7	A	A	C	T	C	0.0365	0.0449	1.32 (0.68 - 2.55)	0.41
8	G	A	C	T	C	0.0365	0.0339	1.12 (0.56 - 2.25)	0.75

a Analyzed by SNPStats program. Haplotypes with frequency < 0.03 in both cases and controls were excluded

b Global haplotype association *P-*value = 0.023

c Boldface means association with increased susceptibility of lung cancer

## DISCUSSION

We have previously identified a susceptibility region on chromosome 19q13.3 in relation to lung cancer risk using a HapMap-based strategy among Chinese (16 tag SNPs) [10]. Fine-mapping studies now enable us to narrow down the set of candidate causal polymorphisms. This study is an elaboration of our previous association analysis with lung cancer including 18 tag SNPs and 14 non-tag SNPs. To the best of our knowledge, this is the first comprehensive fine-mapping of lung cancer (or cancer) susceptibility encompassing *ERCC2*, *PPP1R13L*, *CD3EAP* and *ERCC1* on chromosome 19q13.3 among Chinese (also Asian).

We have previously reported that *PPP1R13L* rs1970764 in intron8 and *CD3EAP* rs967591 in the 5′ UTR and *CD3EAP* rs735482 in exon3 were associated with increased lung cancer risk in a co-dominant model or dominant model after adjustment for smoking duration [11, 10]. In the present study, we report that rs1970764 was associated with lung cancer risk in a recessive model, and rs967591 and rs735482 was associated with lung cancer risk in a log-additive model as previously reported (Table 2). In addition, we identified 4 new polymorphisms in the vicinity of three SNPs as being associated with lung cancer risk. There are *PPP1R13L* rs1005165 near the 5′ end of *CD3EAP, CD3EAP* rs1007616 in the 3′ UTR, rs62109563 in the 3′ UTR, and *ERCC1* rs3212965 in intron5.

We determined 6 haplotype blocks in the sub-region of chromosome 19q13.3. Seven SNPs which were associated with lung cancer risk were partitioned in block 3 and block 5. *PPP1R13L* rs1970764 is located in block 3. The remaining 6 SNPs were all located in block 5. The 6 SNPs were in strong pair-wise linkage disequlibrim with one another (all D' > 0.8) (Figure 1), implying that they probably detect the same biological effect. Both *PPP1R13L* rs1970764 in block 3 and *CD3EAP* rs967591 in block 5 were important constituents of the previously identified “high-risk haplotype” associated with increased risk of several cancers among Caucasian [12–14]. We also proposed the two SNPs as risk candidates for single genetic locus among Chinese [11, 15]. Recent studies of Koreans reported that *PPP1R13L* rs1970764 was significantly associated with relapse-free and disease-specific survival in a recessive model for rectal cancer and *CD3EAP* rs967591 AA genotype exhibited lower overall survival of early-stage lung cancer [16, 17]. The two blocks spanning *ERCC2*, *PPP1R13L*, *CD3EAP* and *ERCC1* within chromosome 19q13.3 were high linkage disequilibrium as also observed in Caucasian Danes [1].

We attempted to locate the causal sequences and polymorphisms. The haplotype block analysis suggested that the causal genetic variation could locate to the 28.406 kb from *ERCC2* intron11 rs238403 to *PPP1R13L* intron8 rs34231843 in block 3 and to the 12.836kb from *PPP1R13L* intron1 rs4803817 to *ERCC1* intron5 rs3212964 in block 5. However, when combining this information with the multiple single marker analysis, our data clearly indicated that the biologically relevant effectors probably locates to the 29.707kb spanning from *PPP1R13L* intron8 rs1970764 to *ERCC1* intron5 rs3212965 as all significant *P*-values were found in this region. The most probable location is therefore in the 23.173kb spanning from *PPP1R13L* intron8 rs1970764 to rs62109563 3′ to *CD3EAP*. This DNA segment contains 6 SNPs with significant *P*-values. The sub-region span was very similar to the region identified for Caucasian Danes [1, 5, 6]. Using an alternative approach where we performed a haplotype analysis of all significant polymorphisms, rs1970764 was found to be most consistently associated with lung cancer risk.

However, the present case-control study group had a modest sample size and verification should be attempted in larger population-based cohorts.

We performed functional predictions for the 7 SNPs that were significantly associated with lung cancer risk using web-based SNP selection tools: SNPinfo [18] and Polyphen-2 [19]. The SNPinfo analysis suggested that rs1005165 at 5′ near gene and rs967591 at 5′ UTR may potentially modify activity of both *PPP1R13L* and *CD3EAP* because of the close proximity of both genes. Rs1005165 with high regulatory potential score (RPS) (0.405099) and conservation score (0.781) and rs967591 with RPS (0.199765) were predicted to create TFBS (Transcription Factor Binding Sites) or TFBS and Splicing [ESE or ESS (Exonic Splicing Enhancer or Exonic Splicing Silencer)]. Rs1007616 in the 3′ UTR and rs62109563 near the 3′ may potentially influence both *CD3EAP* and *ERCC1* since the two SNPs locate to a region with overlapping transcription of *CD3EAP* and *ERCC1*. TFBS and MicroRNA-binding sites were predicted for rs1007616. A benign influence was predicted by Polyphen-2 for rs735482 which is a non-synonymous SNP. Thus, 4 of the SNPs associated with lung cancer risk could potentially be the biologically relevant polymorphism. An important next step would be to experimentally characterize the potential biological effects of these all candidate SNPs.

In summary, we have fine-mapped the sub-region encompassing *ERCC2*, *PPP1R1*, C*D3EAP* and *ERCC1* on chromosome 19q13.3 region in relation to lung cancer susceptibility among Chinese. We observed that of the 32 (39) SNPs directly genotyped covering a 71.654kb region, seven closely situated SNPs were all associated with lung cancer risk. Studying combinations of markers, LD blocks and their haplotypes, we suggest that a sub-region with the strongest association to lung cancer susceptibility might locate to the 23.173kb from *PPP1R13L* intron8 rs1970764 to rs62109563 3′ to *CD3EAP*. Further fine-mapping studies of cancer susceptibility with other Asian populations should focus on these loci.

## MATERIALS AND METHODS

### Ethics statement

The Chinese Administration Office of Human Genetic Resources approved this protocol. It complied with the principles outlined in the Helsinki Declaration. All study participants granted written or oral informed consent.

### Samples

The studied population comprised 771 subjects, including 384 cases with lung cancer and 387 cancer-free controls using the same study population with increased sample size [10]. Briefly, lung cancer diagnosis was based on standard clinical and histological criteria. Eligible cases were previously untreated (recruited prior to chemotherapy or radiotherapy for cancer). Cancer-free controls were identified from the orthopedics wards in the same region. Cancer-free status was ensured by Doctor's query in detail. Cancer-free, randomly selected controls were matched to the cases by ±3 years age, sex and ethnicity (So population stratification was not carried out). All subjects were unrelated ethnic Han Chinese. All covariate data were obtained from questionnaires. Stratification analyses were defined by gender, age (10-year intervals) and smoking history (20-year intervals) (Table 1).

### SNP selection

We added more non-tag SNPs in present study to tag SNPs from our previous studies and combined all data to enable fine-mapping of lung susceptibility on chromosome 19q13.3. As a whole, SNPs were determined in the Chr19q13.3 lung cancer candidate region spanning 45855262 - 45926916 bp (range: 71.654kb) from 37.3 Genome Build in NCBI dbSNP. In total 39 SNPs, containing 18 tag SNPs (r^2^ ≥ 0.80 and MAF ≥ 0.05) and 21 non-tag SNPs with MAF ≥ 0.05, were included to capture the variability present across the sub-region in Chinese populations. The tag SNPs covered 90% of the common variation in the sub-region. The risk loci from our previous publications [10] were used as the center source of selection of non-tag SNPs. More details are presented in Table 4.

**Table 4:** The information of selected 39 SNPs in *ERCC2, PPP1R13L, CD3EAP* and *ERCC1* on Chr19q13.3

Gene/dbSNP ID	Location	Position	Base change	Allele frequency in HapMap CHB	MAF in control group	Genotyping technique
***ERCC2***^a^						
rs238418	intron	45855262	C/T	C0.675/A0.325	A0.33	Sequenom
rs1799787/Tag	intron	45856144	C/T	C0.932/T0.068	T0.06	PCR-RFLP
rs3916874/Tag	intron	45856926	G/C	G0.811/C0.189	C0.17	PCR-RFLP
rs238415/Tag	intron	45857235	C/G	C0.525/G0.475	G0.45	Sequenom
rs238414	intron	45857820	C/T	C0.608/T0.392^b^	T0.48	Sequenom
rs2070831	intron	45858246	C/T	C0.767/T0.233^b^	T 0.27	Sequenom
rs3916840/Tag	intron	45862297	C/T	C0.944/T0.056	T 0.04	PCR-RFLP
rs50872/Tag	intron	45862449	C/T	C0.709/T0.291	T0.19	Sequenom
rs50871/Tag	intron	45862515	T/G	T0.678/G0.322	G0.25	Tag-Man
rs238403/Tag	intron11	45865217	C/T	C0.560/T0.440	T0.47	PCR-RFLP
rs2097215^c^	5′ near gene	45875787	A/G	A0.537G0.463	G0.49	Sequenom
*****PPP1R13L***** ^a^						
rs6966/Tag	3′ UTR	45882962	A/T	A0.500/T0.500	T0.48	PCR-RFLP
rs8112723^c^	intron	45885279	A/T	A0.533/T0.467^b^	T0.31	Sequenom
rs201704^c^	intron	45887265	A/T	No	T0.49	Sequenom
rs2070830/Tag	intron	45889650	G/T	G0.667/T0.333	T0.28	Sequenom
rs1970764	intron8	45890873	A/G	No	G0.46	LDR-PCR
rs10418623^d^	intron	45891670	G/A	G0.667/A0.333^b^	0	Sequenom
rs35209357	intron	45892719	G/C	G0.692/C0.308^b^	C0.3	Sequenom
rs34231843	intron8	45893623	A/G	A0.692/G0.308^b^	G0.29	Sequenom
rs4802252/Tag	intron	45904759	C/T	C0.732/T0.268^b^	T0.24	LDR-PCR
rs4803816	intron	45904888	T/C	T0.707/C0.293	C0.24	Sequenom
rs4803817/Tag	intron1	45907960	A/G	A0.678/G 0.322	G0.34	LDR-PCR
rs1005165	5′ near gene	45909050	C/T	C0.575/T0.425	T0.43	Sequenom
*****CD3EAP***** ^a^						
rs967591	5′ UTR	45909934	G/A	G0.525/A0.475^b^	A0.39	LDR-PCR
rs8113779	intron	45910003	G/T	G0.517/T0.483^b^	T0.43	Sequenom
rs1046282/Tag	intron/3′ UTR for ERCC1	45910672	T/C	T0.673/C0.327^b^	C0.34	LDR-PCR
rs735482/Tag	exon3/3′ UTR for *ERCC1*	45912002	A/C	A0.556/C0.444	C0.44	LDR-PCR
rs3212986^c^	exon3/3′ UTR for *ERCC1*	45912736	G/T	G0.686/T0.314	T0.33	Sequenom
rs1007616	3′ UTR	45913093	C/T	C0.683/T0.317^b^	T0.25	Sequenom
rs62109563	3′ near gene	45914046	T/C	T0.650/C 0.350^b^	C0.35	Sequenom
*****ERCC1***** ^a^						
rs3212980/Tag	intron		A/C	A0.679/C0.321^b^	C0.34	LDR-PCR
rs3212967^d^	intron	45920264	C/T	C0.550/T0.450^b^	T1.0	Sequenom
rs3212965	intron5	45920580	C/T	C0.689 /T0.311	T0.33	Sequenom
rs3212964/Tag	intron5	45920796	G/A	G0.567/A0.433	A0.43	LDR-PCR
rs3212961/Tag	intron	45922323	A/C	A0.500/C0.500	A0.48	LDR-PCR
rs3212955	intron	45923496	A/G	A0.711/G0.289	G0.29	Sequenom
rs11615/Tag	exon4	45923653	G/A	G0.778/A0.222	A0.23	PCR-RFLP
rs3212950^d^	intron	45924086	C/G	C0.658/G0.342^b^	C1.0	Sequenom
rs2298881/Tag	intron	45926916	T/C	T0.517/A0.444	A0.4	LDR-PCR

a The information from NCBI SNP database (37.3 Genome Build) and HapMap database

b CHB+JPT

c Hardy-Weinberg equilibrium departure

d Genotyping fail

### Genotyping platform

Sequenom MassARRAY iPLEX platform (San Diego, CA, USA) was used for genotyping of *ERCC2* rs238418, rs238414, rs2070831, rs50872, rs2097215; *PPP1R13L* rs8112723, rs201704, rs10418623, rs35209357, rs34231843, rs4803816, rs1005165; *CD3EAP* rs8113779, rs3212986, rs1007616, rs62109563; and *ERCC1* rs3212967, rs3212965, rs3212955, rs3212950. Genotypes of *ERCC2* rs1799787, rs3916874, rs3916840, rs50871, rs238403; *PPP1R13L* rs6966, rs1970764, rs4802252, rs4803817; *CD3EAP* rs967591, rs1046282, rs735482; and *ERCC1* rs3212980, rs3212964, rs3212961, rs11615, rs2298881 were determined by methods of PCR-RFLP or Taq-Man or LDR-PCR as our previous reports [7–11]. Assay design and mass spectrometric genotyping were performed as previously described [20] with modifications as indicated in Supplementary Material, Methods. Primer and probe sequences are listed in Supplementary Material, Table 2. As previously described, assay design failed for rs50872 of *ERCC2* [7], whereas the genotype distribution of rs2070830 in *PPP1R13L* (strongly) [9] and rs238415 in *ERCC2* (slightly) [7] deviated from Hardy-Weinberg equilibrium among the controls. The three SNPs were regenotyped using Sequenom platform in the present analysis.

### Statistics methods

For each SNP, Hardy-Weinberg equilibrium test, allele frequencies, and genotype frequencies were calculated using the SNPStats program [21] and Plink software v1.07 (http://pngu.mgh.harvard.edu/~purcell/plink/). Genotype distribution for each SNP among controls was tested for deviation from Hardy-Weinberg equilibrium and rejected at *P* < 0.05. Unconditional logistic regression was applied for calculation of adjusted OR, adjusted for smoking duration) and interaction between genotypes and smoking duration by using SNPStats. We did not adjust for family history of cancer since the study object is genetic susceptibility factors. Five genetic models (co-dominant model, dominant model, recessive model, over-dominant model and log-additive model) (Table 2) were performed for each single-locus case-control association. Haploview software 4.2 [22] and SNPStats program [21] were used to calculate D' and r^2^ values between the genotyped SNPs and haplotype frequencies, to generate D' and r^2^ map and LD block boundaries (based on 95% confidence bounds on D' values [23]) and to analyze the haplotype associations (OR,, adjusted for duration of smoking) of LD blocks identified. Haplotypes with frequency < 0.03 among both cases and controls were excluded from the analysis.

## SUPPLEMENTARY MATERIALS FIGURES AND TABLES


