# Supplementary material for: Fine-mapping markers of lung cancer susceptibility in a sub-region of chromosome 19q13.3 among Chinese
Source: Oncotarget. 2016 May 10;7(38):60929–39. doi: 10.18632/oncotarget.9279 (PMC5308627; doi:10.18632/oncotarget.9279)
Supplement: Supplementary file 1 [file oncotarget-07-60929-s001.pdf]

Fine-mapping markers of lung cancer susceptibility in a sub-region of chromosome 19q13.3 among Chinese

Supplementary Material

Supplementary Methods

Assay of Sequenom SNP

Whole assay was performed according to the manufacturer's recommendations [iPLEX® Gold Application Guide (July 22, 2009), SEQUENOM]

1. Design and synthesis of primers and probes
   Design software: AssayDesigner3.1.
   Synthesis Company: Invitrogen Biotechnology Co., Ltd., Beijing, China.

2. DNA quality test and working dilution
   Confirm eligibility DNA by OD test and agarose electrophoresis.
   Standard concentration of eligibility DNA: 20～30ng/μl.

3. PCR amplifying
   Reagents: Sequenom
   Company. PCR instrument:
   ABI veriti-384.

   Reaction cocktail：

Reagents	Volume of Reagents in 5μl
Water, HPLC grade	1.8
10 x PCR Buffer	0.5
25mM MgCl2	0.4
25mM dNTP Mix	0.1
0.5uM Primer Mix	1
5U/μl Taq	0.2
20ng/μl DNA	1
Final volume [μl]	5

Procedure of thermocyling PCR reactions:

1	94 ℃	15 min	
2	94 ℃<br>56 ℃<br>72 ℃	20 sec<br>30 sec<br>1 min	45 cycles
3	72 ℃<br>4℃	3 min<br>∞	

4. SAP (Shrimp Alkaline Phophatse) reaction-Neutralizing unincorporated dNTPs
Reagents: Sequenom Company.
PCR instrument: ABI veriti-384.

Reaction cocktail：

Reagents	Volume of reagents in 7μl of SAP cocktail
Nanopure water, autoclaved	1.53
SAP Buffer	0.17
SAP Enzyme (1.7U/μl)	0.3
PCR reaction products	5
Final volume [μl]	7

Procedure of SAP reaction:

37 ℃ 40 min, 85 ℃ 5 min, 4 ℃ ∞

5. Extend reaction-Creating the iPLEX gold reaction
Reagents: Sequenom Company.
PCR instrument: ABI veriti-384.

Reaction cocktail:

Reagents	Volume of reagents in 9μl of extend cocktail
Nanopure water, autoclaved	0.619
iPLEX Buffer Plus	0.2
iPLEX Termination mix	0.2
iPLEX Extend Primer Mix	0.94
iPLEX Enzyme	0.041
Volume [μl]	2
SAP+PCR reaction products	7
Total Volume [μl]	9

Procedure of extend reaction:

1	94 ℃	30 sec		
2	94 ℃	5 sec		40 cycles
	52 ℃	5 sec	5 cycles	
	80 ℃	5 sec		
3	72 ℃	3 min		
	4℃	∞		

6. Conditioning the iPLEX gold reaction products
   Remove cation component by resin.

7. Dispensing onto SpectroCHIP Arrays

8. Defining assays and plates

9. Acquiring and analyzing spectra
   Acquire spectra using the MassARRAY mass spectrometer (Sequenom) and analyze using TyperAnalyzer Software 4.0 (Sequenom).

Supplementary Table 1: Haplotype analyses of linkage disequilibrium blocks identified, adjusted by smoking duration

Haplotype structure[a,b]	Case frequency[a]	Control frequency[a]	OR (95%CI)[b]	*P*-value[b]
**Block 1** (P^c = 0.68). **SNPs**: ***ERCC2*** rs238418(C>A), rs1799787(C>T)				
CC	0.663	0.666	1.0	
AC	0.271	0.274	1.02 (0.80 - 1.30)	0.86
AT	0.066	0.058	1.18 (0.75 - 1.87)	0.47
**Block 2** (P^c = 0.68). **SNPs**: ***ERCC2*** rs3916874(G>C), rs238415(C>G), rs238414(C>T), rs2070831(C>T), ***PPP1R13L*** rs 6966(A>T)				
GGCC	0.460	0.447	1.0	
GCTT	0.264	0.269	0.97 (0.75 - 1.25)	0.8
CCTC	0.153	0.174	0.81 (0.60 - 1.09)	0.16
GCCC	0.083	0.070	1.16 (0.77 - 1.74)	0.47
GCTC	0.034	0.036	1.06 (0.57 - 1.96)	0.86
**Block 3** (P^c = 0.61). **SNPs**: ***ERCC2*** rs238403(C>T), ***PPP1R13L*** rs 6966(A>T), rs2070830(G>T), rs1970764(A>G), rs35209357(G>C), rs34231843(A>G)				
TAGAGA	0.437	0.451	1.0	
CTTGCG	0.270	0.249	1.22 (0.94 - 1.60)	0.14
CTGGGA	0.183	0.178	1.04 (0.77 - 1.40)	0.79
CAGAGA	0.043	0.045	0.89 (0.50 - 1.59)	0.69
**Block 4** (P^c = 0.11). **SNPs**: ***PPP1R13L*** rs4802252(C>T), rs4803816(T>C)				
CT	0.780	0.749	1.0	
TC	0.214	0.236	0.83 (0.65 - 1.06)	0.13
**Block 5** (**P^c = 0.011**). **SNPs**: ***PPP1R13L*** rs4803817(A>G), rs1005165(C>T), ***CD3EAP*** rs967591(G>A), rs8113779(G>T), rs1046282(T>C), rs735482(A>C), rs1007616(C>T), rs62109563(T>C), ***ERCC1*** rs3212980(A>C), rs3212965(C>T), rs3212964(G>A)				
ATATTCCCACA	0.342	0.294	1.0	
GCGGCACTCTG	0.284	0.310	0.82 (0.62 - 1.08)	0.15
ACGGTATTACG	0.198	0.219	**0.72 (0.53 - 0.97)**[d]	**0.032**[d]
ATATTCCTACA	0.076	0.071	0.88 (0.57 - 1.37)	0.58
ATGTTCCCACA	0.020	0.036	0.58 (0.30 - 1.12)	0.11
**Block 6** (P^c = 0.29). **SNPs**: ***ERCC1*** rs11615(G>A), rs2298881(A>C)				
GA	0.430	0.401	1.0	
GC	0.351	0.366	0.86 (0.66 - 1.11)	0.24
AC	0.214	0.230	0.81 (0.61 - 1.07)	0.13

[a] Analyzed by Haploview software 4.2. Haplotypes with frequency < 0.03 in both cases and controls were excluded

[b] Analyzed by SNPStats program, adjusted by smoking duration

[c] *P* for global haplotype association in the block between cases and controls

[d] Boldface means association with decreased susceptibility of lung cancer

Supplementary Table 2: The sequences (5′-3′) of primers and probes for 22 SNPs examined by Sequenom MassARRAY

Gene and rs number	Primers	Probes
ERCC2 rs238418	F: ACGTTGGATGATTCGAGGGGCCACAGATG<br>R: ACGTTGGATGTCTCCTGAAAACGCCCAATG	ACTTCTCTCACCCTGCC
rs238415	F: ACGTTGGATGGCAAAGGTGTCTTAAGTAGG<br>R: ACGTTGGATGTCGGCCTTGTGCTTCAATAG	CTGTTACCCAGTCCCCACAGC
rs238414	F: ACGTTGGATGTCTGCATTCTCAGCCTGATG<br>R: ACGTTGGATGGTGCAGCATGTAGGAATGGG	ACACACCCCCATGCC
rs2070831	F: ACGTTGGATGAGGTGTGACTTCAGGAAGTG<br>R: ACGTTGGATGGGCTTTTCACACATATCCCC	GACCTCCTCCTCCTCCC
rs50872	F: ACGTTGGATGTTCCTGGAGGACAAGACATC<br>R: ACGTTGGATGTTCCTCATCCCCCAACCAC	CCCTCCCCCTCATCCTTAGG
rs2097215	F: ACGTTGGATGAGACTCCGTCCCAGAAAAAG<br>R: ACGTTGGATGATCACTTGGCCAACACTCAC	ATTGGACAGTAGACATCCTGTCAT
PPP1R13L rs8112723	F: ACGTTGGATGCCTGTAATCCCAGCACTTTG<br>R: ACGTTGGATGCCAGGCTGGTTTTGATCTAC	TTTGATCTACTGACCTCAAA
rs201704	F: ACGTTGGATGCCAATAGACCTGCAGCTGAG<br>R: ACGTTGGATGTGGTGTAGATGTCCTTTGTG	GGGTATGTTTGTTCATTTTCCTTCTAAC
rs2070830	F: ACGTTGGATGCATAGACAGGGAATCCTGTG<br>R: ACGTTGGATGCCAGCTTTCTCCCAAGTTTC	TAGTTTCAGGGCCCAA
rs10418623	F: ACGTTGGATGATGCCTGGTTTGAGACCAGC<br>R: ACGTTGGATGCACCACCACTCCCAGATAAT	CCTCCCAGATAATATTTAACATTTT
rs35209357	F: CGTTGGATGAAGATCAGCAGGAGACCATC<br>R: ACGTTGGATGCTTAGCATAATACCTGGCAC	ACTGTTAACTGCTCTTACT
rs34231843	F: ACGTTGGATGTTAACCACCAGGACCAGATG<br>R: ACGTTGGATGAGGGAGAAAGCAAAACGCTG	AGAAGTACGATAAATAGCTAGA
rs4803816	F: ACGTTGGATGCTGGGTTAGACAAATTGGAG<br>R: ACGTTGGATGCCTAAACCCCTCAGCTAAAG	GCCTATTGTTGGAAAGTT
rs1005165	F: ACGTTGGATGACCCACTTCCCTTCCCACTG<br>R: ACGTTGGATGACTGGCCAGGAATGCAGTCG	ATGCAGTCGGGTCAC
CD3EAP rs8113779	F: ACGTTGGATGCAGGATGGAGGAGCCCCAG<br>R: ACGTTGGATGCCCTTTCTCCTTCCACCAAC	GTGCCACCAACGCACCCT
rs3212986	F: ACGTTGGATGCTTTAGTTCCTCAGTTTCCC<br>R: ACGTTGGATGCACAGGCCGGGACAAGAAG	AAAAGGCCGGGACAAGAAGCGGAAG
rs1007616	F: ACGTTGGATGCTGGGTAAATCTAGAGTGGG<br>R: ACGTTGGATGACTGGATTGTTTGTAACTC	GGGATGATTGTTTGTAACTCAATGGATA
rs62109563	F: ACGTTGGATGCATGAGATCCTGTCAATTAC<br>R: ACGTTGGATGCTGTGCCTGGCTTATTTCAC	TAACATAATAATCCCCAGTTCA
ERCC1 rs3212967	F: ACGTTGGATGCTGTAATCCCAGCTACTAGG<br>R: ACGTTGGATGGATCTTGGCTCACTGCAACG	GGCTTCCTCCCGGGTTCAAGCAGTTC

rs3212965	F: ACGTTGGATGACCTGTCCCCAGACACTGAT<br>R: ACGTTGGATGAAGAAGCCCTCCCTGATCC	CGCAGCCCTGGCCACT
rs3212955	F: ACGTTGGATGAACACAGGGTCCCACCAAG<br>R: ACGTTGGATGAGGGTCATGTCCCAGTGTTC	AGGTGTTCTGGACTGTTCT
rs3212950	F: ACGTTGGATGGTCAGGAGTTCGAGACCAG<br>R: ACGTTGGATGTGAACACTTCCTGCCCTCAC	CGGAGAGATGGGGTGTCACCATATT

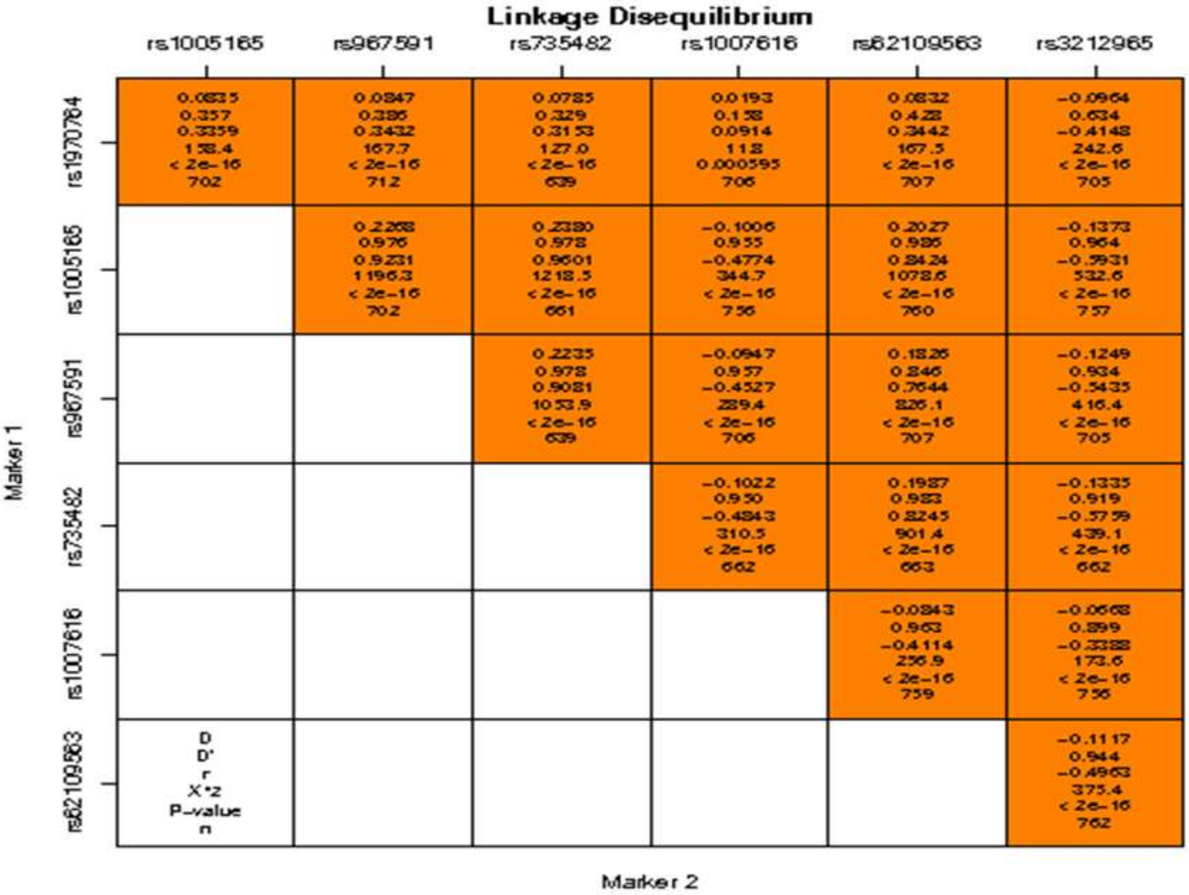

Supplementary Figure 1: Linkage disequilibrium analysis of the seven polymorphisms with statistically significant association with lung cancer risk (from 3′ to 5′: rs1970764, rs1005165, rs967591, rs735482, rs1007616, rs62109563, rs3212965). The figure was generated using the SNPStats program.
